# The Interplay Between Oxidative Stress and Lipid Composition in Obesity-Induced Inflammation: Antioxidants as Therapeutic Agents in Metabolic Diseases

**DOI:** 10.3390/ijms26178544

**Published:** 2025-09-02

**Authors:** Mariló Olivares-Vicente, María Herranz-López

**Affiliations:** Instituto de Investigación, Desarrollo e Innovación en Biotecnología Sanitaria de Elche (IDiBE), Miguel Hernández University (UMH), 03202 Elche, Spain; maria.olivaresv@umh.es

**Keywords:** oxidative stress, obesity, adipocyte hypertrophy, lipid intermediates, inflammation, insulin resistance, antioxidants

## Abstract

Adipose tissue functions not only as a lipid storage depot but also as an active endocrine organ that regulates key physiological processes. In obesity, oxidative stress disrupts the molecular pathways for adipose tissue homeostasis, triggering chronic inflammation, tissue dysfunction, and metabolic disorders. This review explores the mechanisms by which lipid storage drives adipose tissue expansion, highlighting the detrimental effects of hypertrophy in promoting oxidative stress, inflammation, and insulin resistance. These processes can ultimately contribute to metabolic pathologies such as cardiovascular diseases and type 2 diabetes. We also discuss how lipid composition influences these pathways, acting as signaling molecules that activate inflammatory and oxidative stress-related signaling cascades. Additionally, we compile evidence from studies on individuals with obesity, identifying lipids, oxidative stress markers, and inflammatory mediators as potential biomarkers of metabolic dysfunction. Finally, we assess the therapeutic potential of antioxidants in mitigating the metabolic effects of obesity, focusing on their mechanisms of actions. By integrating these insights, this review aims to clarify the complex relationship between oxidative stress, lipid metabolism, and inflammation, and highlight the role of antioxidant molecules in addressing adipose tissue dysfunction in obesity.

## 1. Introduction

The prevalence of obesity has risen substantially in recent decades. In 2022, it was estimated that 504 million women and 374 million men were living with obesity, reflecting increases of 9.7% and 9.2%, respectively, since 1990 [1]. Future projections indicate that this trend is likely to persist, presenting significant challenges for public health. Obesity is defined as a complex, multifactorial, and chronic disease characterized by an excessive accumulation of body fat, typically resulting from a chronic imbalance between caloric intake and energy expenditure. This excess adiposity is associated with a cluster of metabolic abnormalities, often referred to as metabolic syndrome, including dyslipidemia, hypertension, low-grade systemic inflammation, and insulin resistance [2]. These alterations substantially increase the risk of developing severe comorbidities such as metabolic dysfunction-associated steatotic liver disease (MASLD), type 2 diabetes mellitus (T2DM), and cardiovascular diseases [2].

Adipose tissue plays a fundamental role in energy homeostasis, acting as the primary depot for the storage of triglycerides derived from both dietary intake and de novo lipogenesis. However, when lipid accumulation exceeds the storage capacity of adipocytes, a cascade of metabolic alterations is triggered, culminating in adipose tissue dysfunction and the dysregulation of lipid homeostasis [3]. Although the underlying mechanisms have not yet been fully elucidated, emerging evidence suggests that reactive oxygen species (ROS) act as crucial mediators in this dysfunction [4]. Lipid overload and the increase in oxidative processes within the adipocyte induce a state of oxidative stress, which interferes with various intracellular signaling pathways, promoting chronic inflammation, insulin resistance, and intrinsic adipose tissue dysfunction.

Furthermore, the alteration of redox processes in obese adipose tissue is linked to modifications in lipid composition, generating bioactive lipid intermediates [5]. These signaling molecules exert pleiotropic effects by modulating inflammatory and insulin signaling pathways, exacerbating the pro-inflammatory state and insulin resistance [6,7]. In this context, the comprehensive identification and characterization of the oxidative, lipid, and inflammatory mediators involved are essential for the accurate diagnosis of the metabolic status in individuals with obesity and for the development of more personalized and effective therapeutic strategies.

Although promoting a healthy lifestyle, based on a balanced diet and regular physical exercise, constitutes a fundamental strategy [8], the multifactorial nature of obesity necessitates the exploration of complementary therapies. Considering the central role of ROS and oxidative stress in the pathogenesis of metabolic alterations associated with obesity, antioxidant supplementation has been extensively explored as a potential therapeutic approach [9].

This review explores how adipocyte hypertrophy in obesity promotes oxidative stress and the subsequent interplay between oxidative stress and altered lipid composition in the pathogenesis of chronic inflammation and metabolic dysfunction. Furthermore, it will discuss the identification of relevant lipids, oxidative markers, and inflammatory mediators as potential biomarkers in obese humans, and finally, examine the therapeutic potential of antioxidants to mitigate these metabolic complications.

To compile this review, we conducted a targeted literature search using databases such as PubMed, Scopus, and Google Scholar. Keywords related to obesity, adipocyte hypertrophy, oxidative stress, ROS, oxidative markers, lipidomics, lipid intermediates, inflammation, inflammatory mediators, insulin resistance, antioxidants, and polyphenols were used in various combinations. We prioritized peer-reviewed original research articles and comprehensive reviews that included in vitro, animal, and human studies. Most references are from the last decade, supplemented by foundational or highly impactful older works for a complete perspective.

## 2. Metabolic and Endocrine Functions of Adipose Tissue

Adipose tissue plays a central role in maintaining systemic energy homeostasis and endocrine signaling. As the primary energy depot, it stores triacylglycerols (TAGs) and releases fatty acids to peripheral tissues during fasting or increased energy demands. Adipocytes, the primary cell type of adipose tissue, are specialized for the storage of lipids in the form of large, unilocular lipid droplets. Their dynamic regulation of lipid metabolism relies on the interplay between lipogenesis and lipolysis.

During lipogenesis, adipocytes primarily acquire dietary fatty acids through the action of lipoprotein lipase (LPL), which hydrolyzes TAGs carried by chylomicrons and very-low-density lipoproteins (VLDLs) [10,11]. Simultaneously, glucose uptake, facilitated by the insulin-responsive transporter GLUT4, provides glycerol-3-phosphate for TAG esterification, a process catalyzed by diacylglycerol acyltransferase (DGAT) [12]. In addition, fatty acids can also be synthesized via de novo lipogenesis (DNL) [13], where acetyl-CoA is converted into malonyl-CoA by acetyl-CoA carboxylase (ACC) and then into palmitate via fatty acid synthase (FAS). DNL is transcriptionally regulated by carbohydrate response element-binding protein (ChREBP) and sterol regulatory element-binding protein 1 (SREBP1) [13].

Lipolysis provides fatty acid fuel to peripheral tissues through the sequential hydrolysis of TAGs into glycerol and free fatty acids by the coordinated and regulated action of adipose triglyceride lipase (ATGL), hormone-sensitive lipase (HSL), and monoacylglycerol lipase (MGL) [14]. Noradrenaline, a catecholamine released by the sympathetic nervous system (SNS), is the primary inducer of lipolysis, acting through β-adrenergic receptors and subsequent activation of protein kinase A (PKA) [15].

While adipocytes are the primary cell type responsible for lipid storage within adipose tissue, it is a complex and heterogeneous tissue comprising a significant population of non-mature adipocytes and other cell types. These cells constitute the stromal-vascular fraction (SVF) and include preadipocytes, macrophages, neutrophils, lymphocytes, stem cells, and endothelial cells. These diverse cell populations co-exist and interact to maintain adipose tissue homeostasis and contribute to systemic energy balance.

Indeed, adipocytes, in concert with other adipose tissue cell types, secrete a wide array of bioactive mediators collectively termed adipokines. These signaling molecules, including hormones (e.g., leptin, adiponectin, resistin), lipids (e.g., fatty acids, eicosanoids), inflammatory cytokines (e.g., tumor necrosis factor-alpha [TNF-α], interleukin-6 [IL-6]), exosomes, and microRNAs (miRNAs), exert autocrine, paracrine, and endocrine effects [16,17]. Consequently, they participate in numerous physiological processes, including the regulation of food intake, reproductive function, insulin sensitivity, and immune responses.

It is extensively recognized that dysregulation of the cellular composition within adipose tissue, or alterations in the secretion profiles of these various cell types, significantly contributes to systemic metabolic dysfunction and the pathogenesis of chronic metabolic diseases, including obesity-associated insulin resistance and inflammation [18,19].

## 3. Adipose Tissue Dysfunction: The Role of Reactive Oxygen Species and Oxidative Stress in Obesity

**Figure 1:**
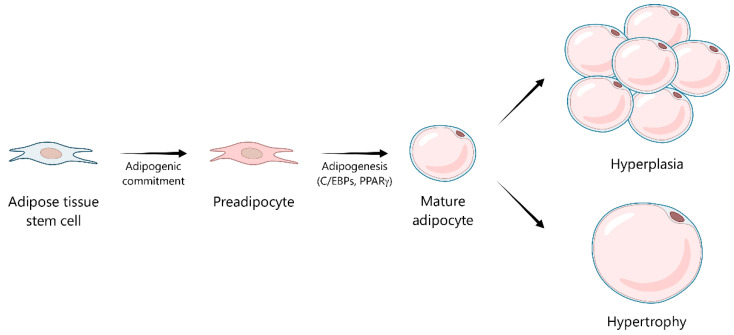
Adipogenesis and adipose tissue expansion. Adipose tissue stem cells commit to the adipogenic lineage, forming preadipocytes. Through the process of adipogenesis, orchestrated by master transcriptional regulators such as the CCAAT/enhancer-binding protein (C/EBP) family and peroxisome proliferator-activated receptor gamma (PPARγ), these preadipocytes differentiate into mature, functional adipocytes. Adipose tissue mass expands via two primary mechanisms: hyperplasia (an increase in the number of adipocytes) or hypertrophy (an increase in the size of existing adipocytes).

In obesity, the expansion of adipose tissue becomes maladaptive, contributing to metabolic dysfunction and increased oxidative stress. Adipose tissue expansion occurs through two primary mechanisms: hyperplasia, involving the generation of new adipocytes from preadipocyte differentiation, and hypertrophy, characterized by an increase in adipocyte size due to lipid accumulation [20] (Figure 1). The contribution of each mechanism is influenced by multiple factors, including the anatomical location of adipose tissue and the age and sex of the individual [21,22]. However, numerous studies have consistently linked adipocyte hypertrophy, particularly in visceral adipose tissue, to metabolic derangements characteristic of obesity, such as chronic low-grade inflammation and insulin resistance [23,24]. Oxidative stress is a major factor driving these metabolic disturbances [25,26].

ROS are unstable molecules due to the presence of unpaired electrons, enabling them to oxidize key cellular components such as lipids, DNA, and proteins. The major ROS include the superoxide anion (O_2_·^−^), hydrogen peroxide (H_2_O_2_), and the hydroxyl radical (·OH). Oxidative stress refers to an imbalance in favor of ROS production over the capacity of cellular antioxidant systems, leading to cellular damage and dysfunction [27].

Nevertheless, when redox balance is maintained, ROS serve as key signaling molecules participating in diverse physiological processes. In adipocytes, insulin-stimulated ROS may enhance insulin signaling by reversibly inhibiting protein tyrosine phosphatases (PTPs) such as PTEN, a lipid and protein phosphatase that negatively regulates the phosphoinositide 3-kinase/protein kinase B (PI3K/Akt) pathway [28]. In preadipocytes, a controlled increase in ROS, particularly H_2_O_2_, facilitates differentiation into mature adipocytes by promoting mitotic clonal expansion and enhancing the activity and expression of key adipogenic transcription factors, such as CCAAT/enhancer-binding protein beta (C/EBPβ) and peroxisome proliferator-activated receptor gamma (PPARγ) [29,30].

However, an imbalance in redox homeostasis within adipose tissue can impair adipocyte function, affecting essential processes such as adipogenesis, promoting insulin resistance, and contributing to adipocyte hypertrophy [31,32]. Adipocyte hypertrophy, in turn, can trigger mitochondrial dysfunction, hypoxia, and endoplasmic reticulum (ER) stress, which collectively promote inflammatory signals and exacerbate ROS production [33,34,35].

The principal intracellular sources of ROS in adipocytes are the nicotinamide adenine dinucleotide phosphate (NADPH) oxidase enzyme family and mitochondria. The NOX family, comprising isoforms NOX1-5 and DUOX1/2, constitutes a group of membrane-bound enzymatic complexes that catalyze the production of O_2_·^−^ or H_2_O_2_ through the reduction in molecular oxygen (O_2_), utilizing NADPH as an electron donor [36]. Among these isoforms, NOX4 has been extensively investigated in adipocytes. Previous studies have shown that, under physiological conditions, NOX4-derived H_2_O_2_ promotes insulin signaling by inhibiting PTP1B, a negative regulator of the insulin receptor [37], and supports adipogenesis in preadipocytes through the activation of MAP kinase phosphatase-1 (MKP-1) [38], which attenuates extracellular signal-regulated kinases 1 and 2 (ERK1/2) signaling. Conversely, during conditions of nutrient excess, such as obesity, NOX4 can be overexpressed and relocalize to lipid rafts within the plasma membrane [39]. This altered expression and localization has been linked to the activation of the pro-inflammatory transcription factor nuclear factor kappa-light-chain-enhancer of activated B cells (NF-κB) and the induction of chemotactic signaling, thereby contributing to inflammation [39].

Notably, the role of NOX4 appears context-dependent. Studies using global NOX4 knockout mice have shown an accelerated development of insulin resistance with a high-fat diet, likely due to a reduction in adipocyte numbers and increased adipocyte hypertrophy [40]. In contrast, deleting NOX4 specifically in adipocytes seems to reduce insulin resistance, possibly by limiting ROS-induced immune cell inflammation [41]. These findings suggest a critical role for this enzyme in maintaining adipose tissue health and proper function.

Mitochondria, essential for adenosine triphosphate (ATP) production, represent another crucial source of ROS in adipocytes, particularly in the context of obesity. The primary origin of mitochondrial ROS is the electron transport chain, with O_2_·^−^ being mainly produced by enzymes in complexes I, III, and, to a lesser extent, II [26]. O_2_·^−^, generated by both mitochondria and NADPH oxidase, is rapidly converted to H_2_O_2_ via dismutation catalyzed primarily by the superoxide dismutase (SOD) enzyme family [42]. Subsequently, H_2_O_2_ can react with metal ions to form the highly reactive ·OH radical through the Fenton reaction. Under physiological conditions, H_2_O_2_ is efficiently detoxified to water by several antioxidant systems, including catalase (CAT), glutathione peroxidases (GPXs), and peroxiredoxins (PRXs), thus limiting ROS accumulation in the cytosol [26,42].

Nevertheless, under conditions of nutrient excess, such as obesity, adipocyte mitochondria undergo notable alterations. The chronic oversupply of glucose and fatty acids increases metabolic flux through glycolysis and the tricarboxylic acid cycle, leading to a higher production of electron carriers, including nicotinamide adenine dinucleotide hydrogen (NADH), and reduced flavin adenine dinucleotide (FADH_2_). This overwhelms the mitochondrial respiratory chain, resulting in increased electron leakage and, consequently, elevated ROS production [4]. Moreover, this sustained metabolic stress disrupts mitochondrial biogenesis, induces changes in mitochondrial number, dynamics and morphology, and reduces mitochondrial DNA content [43,44]. Indeed, obesity is associated with reduced activity of key mitochondrial enzymes. Specifically, the decreased expression of alpha subunit of hydroxyacyl-CoA dehydrogenase (HADHA), citrate synthase, or respiratory chain complex components has been observed in the adipose tissue of individuals with obesity [45,46]. These impairments contribute to diminished β-oxidation and oxidative phosphorylation, further exacerbating ROS production.

Mitochondrial dysfunction may impair key metabolic processes, such as adipogenesis, lipolysis/lipogenesis balance, and adiponectin production [47]. For instance, the inhibition of complex III in 3T3-L1 preadipocytes leads to TAG accumulation and the downregulation of key adipogenic transcription factors such as C/EBPα and C/EBP homologous protein (CHOP-10) [48]. Reduced mitochondrial DNA content in human adipose tissue has been correlated with higher body mass index (BMI) and diminished basal and insulin-stimulated lipogenesis [49]. Furthermore, mitochondria are essential for the synthesis of adiponectin, an anti-inflammatory adipokine that regulates glucose and lipid metabolism and promotes insulin sensitivity. Consistently, studies on obese mice have linked decreased mitochondrial content to lower adiponectin expression [50]. In 3T3-L1 adipocytes, excessive ROS generated by mitochondrial dysfunction impairs adiponectin secretion, thereby contributing to defective insulin signaling and disrupted glucose homeostasis [51].

Furthermore, the redox balance in obese adipose tissue is often disrupted by a dysregulation of antioxidant enzymes. Studies on visceral adipose tissue from individuals with obesity have shown decreased levels of reduced glutathione (GSH), reduced expression of manganese SOD, and increased expression of NADPH oxidase [52]. Similarly, peripheral blood mononuclear cells from patients with obesity exhibit lower activities of SOD, CAT, and GPX, alongside an increased oxidized-to-reduced glutathione ratio (GSSG/GSH) [53]. Nuclear factor erythroid 2-related factor 2 (Nrf2) plays a pivotal role as a master transcriptional regulator of these antioxidant defenses in response to oxidative stress. However, in obesity, Nrf2 appears to be impaired despite increased ROS levels, potentially worsening oxidative damage [54]. This dysregulation highlights Nrf2 as a potential therapeutic target in the management of obesity-related oxidative damage [55].

## 4. The Link Between Oxidative Stress with Inflammation and Insulin Resistance in Obese Adipose Tissue

Obesity-induced oxidative stress is closely linked to adipose tissue inflammation and impaired insulin signaling. ROS generated by glucose and fatty acids activate inflammatory pathways, particularly through the NF-κB signaling pathway, which drives the expression of pro-inflammatory cytokines and chemokines [56,57]. Among these, monocyte chemoattractant protein-1 (MCP-1, also known as CCL2) is a key mediator, directly promoting the recruitment of monocytes and macrophages to adipose tissue [56]. Consistent with this, a previous study demonstrated that NOX4-generated ROS play a pivotal role in mediating the increased production of MCP-1 in adipocytes under conditions of nutrient excess [39].

**Figure 2:**
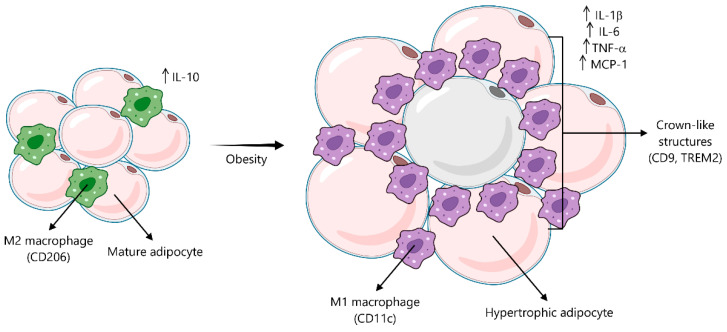
A shifting identity of macrophages in healthy and obese adipose tissue. Healthy adipose tissue is characterized by mature adipocytes and resident M2 macrophages, which are typically identified by markers such as CD206 and establish an anti-inflammatory environment. However, the progression towards obesity leads to significant adipose tissue remodeling, including adipocyte hypertrophy and the recruitment and polarization of macrophages to a pro-inflammatory M1 phenotype, often expressing CD11c. These M1 macrophages frequently cluster around dying adipocytes, forming characteristic crown-like structures (CLSs) and co-expressing distinct markers like CD9 and TREM2.

Obese adipose tissue is characterized by a marked accumulation of macrophages, eventually constituting up to 40% of the SVF [58]. Adipose tissue macrophages (ATMs) are key effector cells driving inflammation and are typically classified into two distinct populations: classical M1 macrophages, which produce pro-inflammatory cytokines, and alternatively activated M2 macrophages, which display an anti-inflammatory profile [59] (Figure 2). Although single-cell RNA sequencing analyses have shown a more complex transcriptomic profile, obese ATMs generally exhibit a pro-inflammatory genetic signature [60]. A significant subset of M1-like macrophages, originating from monocytes, expresses CD11c+ and secretes pro-inflammatory cytokines such as IL-1β and TNF-α, contributing to insulin resistance [61]. Furthermore, CD9+ macrophages accumulate around dying adipocytes, forming the so-called crown-like structures (CLSs) (Figure 2). Within this subpopulation, TREM2-expressing lipid-associated macrophages (Trem2+ CD9+) play a key role in lipid metabolism and metabolic homeostasis [62].

Macrophages have been identified as the main cellular source of the NOX2 isoform in visceral adipose tissue, where NOX2 acts as a key regulator of pro-inflammatory cytokine expression and lysosomal exocytosis in response to dead adipocytes [63]. Previous studies have shown that NOX2-deficient mice fed a high-fat diet exhibit reduced visceral fat accumulation and adipocyte hypertrophy, decreased macrophage infiltration, and improved glucose homeostasis compared to wild-type mice [63]. However, more recent findings have associated prolonged high-fat diet feeding with impaired lysosomal exocytosis of dead adipocytes and the development of severe insulin resistance in the absence of NOX2 [64], suggesting that the effects of NOX2 may depend on the progression of obesity.

ATMs may represent a crucial source of ROS within adipose tissue. The obese microenvironment, characterized by saturated fatty acids (SFAs) from the diet, lipids released by hypertrophic adipocytes, pro-inflammatory cytokines, and circulating lipopolysaccharide (LPS) derived from an altered gut microbiota [65,66,67], activates macrophages and stimulates ROS production, primarily through NOX2 activity and mitochondrial dysfunction. This, in turn, promotes and sustains M1 macrophage polarization, establishing a positive feedback loop that perpetuates adipose tissue inflammation [68,69].

Within the adipose tissue, the elevated levels of ROS also directly activate the NF-κB pathway in the infiltrating macrophages by promoting the degradation of its inhibitor IκBα, leading to the nuclear translocation of NF-κB and the transcription of numerous pro-inflammatory genes, like TNF-α, IL-6, and IL-1β [70,71]. Notably, mitochondrial ROS in macrophages have been shown to promote IκB kinase (IKK) complex activation through the formation of a disulfide bridge on the regulatory subunit NEMO [72], directly linking mitochondrial dysfunction to NF-κB-mediated inflammation. Similarly, adipocyte- and macrophage-derived ROS can activate other critical inflammatory signaling pathways such as the mitogen-activated protein kinase (MAPK) signaling cascade [73], through the oxidation of cysteine residues in MAP kinase kinase kinases (MAPKKKs) [74], or by inactivating MAPK phosphatases (MKPs) [75]. Notably, the c-Jun N-terminal kinases (JNK) pathway is upregulated in obesity and contributes to insulin resistance in adipose tissue [76].

Finally, another crucial inflammatory mechanism influenced by ROS in obesity is the activation of the nucleotide-binding domain, leucine-rich-containing family, pyrin domain-containing-3 (NLRP3) inflammasome [77], a multiprotein complex that drives the activation of caspase-1 and the subsequent cleavage and maturation of pro-inflammatory cytokines such as IL-1β and IL-18. Notably, the components of the NLRP3 inflammasome are expressed in adipose tissue, and their expression is upregulated in obesity and insulin resistance [78]. In this context, mitochondrial ROS and oxidized mitochondrial DNA have been implicated in directly activating the NLRP3 sensor [77,79,80]. In parallel, ROS may promote the dissociation of thioredoxin-interacting protein (TXNIP) from its redox-regulating partner thioredoxin-1, thereby allowing TXNIP to bind and activate NLRP3 [81]. Moreover, NLRP3 activation can further promote ROS production, establishing a feed-forward loop that perpetuates inflammation in obese adipose tissue [79].

The chronic low-grade inflammation orchestrated by ROS-activated pathways in obese adipose tissue plays a central role in the development of insulin resistance. NF-κB and JNK activation impair insulin signaling by promoting the inhibitory serine phosphorylation of insulin receptor substrate-1 (IRS-1) or reducing its expression [82,83,84,85], subsequently blunting the PI3K/Akt signaling cascade crucial for glucose uptake. Moreover, activation of the NLRP3 inflammasome contributes to insulin resistance through the release of IL-1β, which, beyond its systemic effects, acts locally in adipose tissue by suppressing IRS-1 expression via both ERK-dependent and -independent mechanisms and by reducing GLUT4 expression and translocation [86]. The sustained and interconnected activation of these ROS-sensitive inflammatory pathways within the obese adipose tissue microenvironment establishes a chronic pro-inflammatory state that significantly contributes to systemic insulin resistance.

## 5. The Crosstalk Between Lipid Mediators and Oxidative Stress in Adipose Tissue Inflammation

Obesity is characterized not only by enlarged lipid droplets within adipocytes but also by a profound dysregulation of the adipose tissue lipidome, affecting both storage and signaling lipids [5]. This altered lipid remodeling could be intricately tied to oxidative stress, potentially serving as both a product and a promoter of its exacerbation. Lipidomic analyses of human white adipose tissue have revealed compositional changes in lipid droplets in obesity, including an increased abundance of TAGs containing at least one polyunsaturated fatty acid (PUFA) and a decrease in those predominantly composed of SFA or monounsaturated fatty acids (MUFAs) [87]. In this regard, it has been demonstrated that increased unsaturation renders lipid droplets more prone to lipid peroxidation.

High levels of ROS in obese adipose tissue can directly damage various biomolecules, including lipids. Lipid peroxidation is a process whereby free radicals target lipids containing carbon–carbon double bonds, which are abundant in PUFAs, leading to the formation of lipid peroxyl radicals and hydroperoxides. Notably, 4-HNE, a toxic lipid peroxidation byproduct, can modulate several transcription factors, including Nrf2, NF-κB, and PPARγ, and also activates key signaling kinases such as MAPKs, Akt, and protein kinase C (PKC) in various cell types [6] (Table 1). In human subcutaneous preadipocytes, acute 4-HNE treatment induces ROS production and antioxidants enzymes, while chronic exposure impairs adipogenesis through SREBP1 and causes IRS-1dephosphorylation, triggering insulin resistance [88]. Interestingly, 4-HNE can upregulate adiponectin gene expression through PPARγ but subsequently promotes its degradation via the ubiquitin–proteasome system, potentially contributing to the reduced adiponectin levels observed in obesity [89]. Furthermore, 4-HNE accumulation in adipocytes has been shown to induce lipolysis by activating the cAMP/PKA/HSL pathway and inhibiting AMP-activated protein kinase (AMPK), leading to increased FFA efflux into the plasma and contributing to lipotoxicity, dyslipidemia, and insulin resistance in peripheral tissues such as muscle and liver [90] (Table 1).

Conversely, the composition of the free fatty acid pool in the epididymal adipose tissue of rats fed a high-fat diet shows a reduction in specific PUFAs (C22:2, C22:4 and C22:5) and an increase in SFAs such as C18:0 (stearic acid) [91]. SFAs, both from diet or lipolysis, have been identified as key signaling molecules linking obesity to inflammation [92]. In macrophages, accumulated stearic acid induces ER stress via the activation of protein kinase RNA-like ER kinase (PERK) and increased expression of binding immunoglobulin protein (BiP), inositol-requiring enzyme 1 alpha (IRE1α), and the pro-apoptotic factor CHOP (Table 1). This, in turn, contributes to the activation of pro-inflammatory pathways, including JNK and NF-κB [93]. Similarly, in high-fat diet mice, stearic acid has been shown to promote the differentiation of pro-inflammatory CD11c^+^ macrophages through a mechanism dependent on retinoic acid receptor signaling and facilitated by the intracellular fatty acid chaperone, epidermal fatty acid binding protein (E-FABP) (Table 1). This evidence highlights the crucial role of excess SFAs in mediating the pro-inflammatory effects characteristic in obesity [94].

SFAs, particularly palmitic acid, contribute to inflammation and metabolic dysfunction in obese adipose tissue by activating TLR4-mediated signaling pathways [95] (Table 1). Notably, TLR4 deficiency mitigates these pro-inflammatory effects, reducing macrophage infiltration and the expression of NF-κB and MCP-1 [96], which consequently improves insulin sensitivity in high-fat diet-fed mice [95]. Through TLR-dependent mechanisms [97,98,99], palmitic acid activates inflammatory pathways in both macrophages and adipocytes and ROS production via NOX enzymes [39,100,101,102]. This leads to increased pro-inflammatory cytokines (IL-1β, IL-6, IL-8, and TNF-α) and reduced anti-inflammatory mediators (IL-10 and adiponectin) [100,101,103] and impairs insulin sensitivity, primarily through the serine phosphorylation of IRS-1 [104] (Table 1).

Interestingly, the phospholipid composition of human visceral and subcutaneous adipose tissue appears to be altered in obesity and insulin resistance [87,105]. Lipidomic analyses have shown that levels of plasmalogens, particularly long-chain PUFA-containing plasmenyl phosphatidylcholine and 18-carbon acyl chain-containing plasmenyl phosphatidylethanolamine, positively correlate with BMI [87]. The vinyl ether bond in plasmalogens is particularly susceptible to oxidation by ROS, enabling these lipids to function as endogenous antioxidants [106] (Table 1). Therefore, the accumulation of plasmalogens in adipose tissue may represent an early compensatory response to increased oxidative stress, which subsequently declines in later stages, potentially due to a collapse of these protective mechanisms [5].

Other phospholipids such as phosphatidylcholine appear to interfere with inflammatory pathways in obesity. Reduced phosphatidylcholine metabolism has been implicated in anti-inflammatory responses, notably through the attenuation of the NLRP3 inflammasome and the preservation of mitochondrial integrity in macrophages. These effects have been linked to enhanced AMPK-dependent mitophagy and a reduction in the cytosolic release of mitochondrial ROS and oxidized mitochondrial DNA [107] (Table 1). Of note, alterations in membrane phosphatidylcholine saturation have been associated with metabolic disturbances. In this regard, a deficiency of lysophosphatidylcholine acyltransferase 3 (LPCAT3) in adipocytes, which leads to reduced levels of polyunsaturated phosphatidylcholine species, has been shown to enhance insulin sensitivity, potentially by facilitating insulin receptor activation and GLUT4 translocation [108] (Table 1).

Furthermore, the increased oxidative stress in obese adipose tissue promotes the generation of oxidized phospholipids (OxPLs), particularly from the oxidation of PUFAs present in phosphatidylcholines such as PAPC and PLPC [109,110]. Specifically, in the SVF of epididymal white adipose tissue from obese mice, an increased proportion of full-length OxPAPC species relative to truncated forms has been observed [110]. This shift in OxPL profile has functional implications for ATMs. While truncated OxPLs induce the expression of antioxidant genes, full-length OxPLs upregulate pro-inflammatory genes such as *Il1β*, *Il6*, and *Cxcl1* [110] (Table 1). Thus, the relative increase in full-length OxPAPC species in obese adipose tissue may contribute to the pro-inflammatory and metabolically activated phenotype adopted by ATMs in obesity.

Finally, sphingolipids such as ceramides have been strongly associated with obesity-related pathologies [111]. In the white adipose tissue of individuals with obesity and high-fat fed-mice, an increase in ceramide accumulation, particularly Cer 16:0, has been observed [112]. This accumulation is promoted by various stimuli [7,111,113]. Pro-inflammatory cytokines such as TNF-α have been shown to induce ceramide synthesis by increasing the expression and activity of sphingomyelinases [114]. Similarly, SFAs (e.g., palmitic acid) and LPS have been shown to induce ceramide synthesis enzymes, likely through a TLR4–NF-κB-dependent mechanism [115,116]. Elevated ceramide levels contribute to metabolic dysfunction by activating the NLRP3 inflammasome [117], inhibiting Akt activation via atypical PKC (PKCζ) [118], and suppressing both mitochondrial respiration and HSL-mediated lipolysis [119,120] (Table 1). Collectively, these effects exacerbate lipid accumulation, oxidative stress, chronic inflammation, and insulin resistance within adipose tissue.

**Table 1:** Dysregulated lipid classes and their contributions to adipose tissue dysfunction in obesity.

Lipid Class	Characteristics	Molecular Targets	Mechanism of Action	References
Polyunsaturated fatty acids (PUFAs)	Contain carbon–carbon double bonds; abundant in cell membranes; susceptible to lipid peroxidation	Nrf2, NFκB, MAPKs, SREBP1, PPARγ, ubiquitin–proteasome system, PKA, AMPK, Akt, PKC	4-HNE (secondary aldehyde): Induces ROS production and inflammation; impairs adipogenesis through SREBP1; induces insulin resistance through IRS-1 dephosphorylation; upregulates adiponectin gene expression through PPARγ, and its degradation via the ubiquitin–proteasome system; induces lipolysis via cAMP/PKA/HSL pathway and inhibiting AMPK, contributing to FFA efflux.	[6,88,89,90]
Saturated fatty acids (SFAs)	No carbon–carbon double bonds; key signaling molecules	ER stress (via PERK, BiP, IRE1α, CHOP), E-FABP, TLR4, NF-κB, JNK, MAPKs, NLRP3 inflammasome, NOX enzymes	Stearic acid (18:0): Activates JNK and NF-κB via ER stress; increases pro-inflammatory cytokines (TNF-α, IL-6, IL-β, MCP-1); promotes macrophage polarization and differentiation via retinoic acid receptor-signaling and E-FABP. Palmitic acid (C16:0): Activates TLR4-mediated signaling (NF-κB, MAPKs and NLRP3 inflammasome); induces ROS production via NOX enzymes; increases pro-inflammatory cytokines (IL-β, IL-6, IL-8, TNF-α); reduces anti-inflammatory mediators (IL-10, adiponectin); impairs insulin sensitivity through IRS-1 serine phosphorylation.	[39,91,92,93,94,95,96,97,98,99,100,101,102,103,104]
Phospholipids	Glycerol backbone with two fatty acid chains and a phosphate group; primary components of cellular membranes. Plasmalogens: Glycerophospholipids with a vinyl ether bond at the sn-1 position and an ester-linked fatty acid at the sn-2 position	ROS, NLRP3 inflammasome, mitochondria, AMPK, insulin receptor	Plasmalogens: Scavenge and neutralize ROS. Phosphatidylcholine: Reduced PC metabolism attenuates inflammation via NLRP3 attenuation; preserves mitochondrial integrity via AMPK-dependent mitophagy and reduced mitochondrial ROS; enhances insulin sensitivity through insulin receptor activation and GLUT4 translocation. Oxidized phospholipids: Truncated OxPLs induce the expression of antioxidant genes (*Ho1*, *Txnrd1*, *Gclm*); full-length OxPLs upregulate pro-inflammatory genes (*Il1β*, *Il6*, *Cxcl1*).	[5,87,105,106,107,108,109,110]
Sphingolipids	Sphingosine backbone linked to a fatty acid; structural membrane components and signaling molecules	NLRP3 inflammasome, Akt (via atypical PKCζ), mitochondrial respiration, HSL	Ceramides: Activate NLRP3 inflammasome and promote IL-1β secretion; inhibit Akt activation and impair glucose uptake; suppress mitochondrial respiration and HSL-mediated lipolysis.	[7,111,112,113,114,115,116,117,118,119,120]

## 6. Lipid Signatures, Oxidative Markers, and Inflammatory Mediators as Biomarkers in Human Obesity

A wide range of biomarkers, including lipid species, oxidative stress markers, and inflammatory mediators, has been investigated to better characterize the metabolic alterations associated with human obesity. The following sections explore these biomarker categories and their potential clinical utility.

### 6.1. Lipid Signatures in Obesity

As previously discussed, emerging lipidomic studies have shown that obesity is associated with specific changes in lipid profiles, particularly in phospholipid and sphingolipid metabolism. In particular, ceramides have been consistently identified as key markers of metabolic dysfunction [121]. Furthermore, a comprehensive study identified a panel of 15 plasma lipid species, mainly consisting of TAGs and phosphatidylcholines, which accurately differentiate lean individuals from individuals with obesity [122]. These results are consistent at the cellular level, where hyperglycemia profoundly alters the lipid profiles of human adipocytes, which act as hallmark signatures of metabolic dysfunction and obesity. This includes a significant accumulation of TAGs accompanied by alterations in other lipid classes such as phospholipids, ceramides, and sphingolipids, reflecting shifts in specific species such as sphingomyelins and phosphatidylcholines. These lipid alterations are characteristic of insulin resistance and related pathologies [123].

Beyond individual lipid species, certain lipid ratios have emerged as valuable biomarkers of obesity-related metabolic risk. The lysophosphatidylcholine to lysophosphatidylethanolamine ratio (LPC/LPE) has been identified as a strong predictor of insulin resistance in obesity, outperforming traditional clinical markers [124]. Similarly, the ceramide/sphingomyelin ratio correlates significantly with visceral adiposity and independently predicts metabolic syndrome development in longitudinal studies [125].

As a novel lipid species, plasmalogen phospholipids have attracted attention for their potential protective role against oxidative damage associated with obesity. While plasmalogen levels in adipose tissue might increase in response to early oxidative stress, subsequent studies have often observed reduced levels in individuals with established obesity. Furthermore, supplementation in diet-induced obese mice has shown improved insulin sensitivity, suggesting that restoring plasmalogen levels might be beneficial when these protective mechanisms become overwhelmed [5].

A novel approach to lipid biomarkers is compartment-specific lipid alterations associated with obesity. Recent advances in tissue-specific lipidomics have demonstrated distinct profiles between subcutaneous and visceral depots, with the latter showing elevated levels of lipid species that promote inflammation and insulin resistance [126]. Additionally, the accumulation of hepatic ceramides and diacylglycerols has been observed to precede the clinical onset of non-alcoholic fatty liver disease, highlighting their potential as early targets for intervention [127].

### 6.2. Oxidative Markers in Obesity

Consistent with the increased ROS production observed in obesity, lipid peroxidation products have emerged as key markers of obesity-associated oxidative stress. In particular, F2-isoprostanes, products of non-enzymatic oxidation of arachidonic acid, have been validated as reliable markers of systemic oxidative stress in obesity [128,129]. Recent studies have shown that urinary levels of 8-iso-prostaglandin F2α correlate positively with BMI and waist circumference and decrease following weight loss interventions [130]. In addition, 4-HNE has been identified as a mechanistic link between obesity and its complications. Elevated plasma 4-HNE levels are associated with insulin resistance and endothelial dysfunction, whereas weight loss interventions reduce circulating 4-HNE levels, improving metabolic parameters [131,132].

Recent proteomics approaches have identified specific patterns of protein oxidation in obesity, preferentially affecting mitochondrial and metabolic enzymes. Plasma protein carbonyl content predicts the development of T2DM in individuals with obesity independently of traditional risk factors [133]. Moreover, advanced glycation end products (AGEs), formed through the non-enzymatic glycation of proteins, accumulate in obesity due to hyperglycemia and oxidative stress. Non-invasive skin autofluorescence measurements have revealed that skin AGE levels predict cardiovascular events in individuals with obesity over a 5-year follow-up period [134].

Likewise, other recent studies have focused on specific antioxidant enzymes, with decreased erythrocyte GPX and SOD activities serving as early indicators of obesity-related oxidative stress. The GSH/GSSG ratio in peripheral blood mononuclear cells correlates inversely with adiposity and improves following weight loss [135]. Interestingly, this ratio shows greater responsiveness to lifestyle interventions than traditional anthropometric measurements, suggesting utility in monitoring early therapeutic responses.

### 6.3. Inflammatory Markers in Obesity

Among the pro-inflammatory cytokines released by dysfunctional adipose tissue and infiltrating macrophages in obesity, recent research has identified specific inflammatory markers, such as IL-1β and IL-18, that exhibit stronger associations with visceral adiposity and metabolic syndrome components than conventional markers like TNF-α and IL-6 [136]. Recent studies have focused on the adiponectin/leptin ratio as an integrated marker of adipose tissue dysfunction, demonstrating superior predictive value for insulin resistance and cardiovascular risk compared to either adipokine alone. It also predicts weight loss outcomes following bariatric surgery, potentially guiding personalized therapeutic approaches [137,138].

The resolution of inflammation is now recognized as an active, coordinated response mediated by specialized pro-resolving mediators (SPMs) derived from omega-3 PUFAs. Recent lipidomic analyses have revealed deficiencies in resolvins, protectins, and maresins in obesity, contributing to persistent inflammation. In this context, plasma levels of resolvin D1 and protectin D1 correlate inversely with inflammatory markers and insulin resistance in obesity. Nutritional interventions targeting SPM production, like omega-3 supplementation, represent a promising therapeutic approach to reduce inflammatory markers in individuals with obesity [139].

Recent years have witnessed a shift from single-marker approaches to integrated panels combining lipid, oxidative, and inflammatory biomarkers. Advanced machine learning algorithms applied to multi-omics data have yielded promising results in predicting obesity-related complications and treatment responses. The translational potential of obesity biomarkers spans diagnosis, risk stratification, and therapeutic monitoring. Diagnostic applications focus on distinguishing metabolically healthy from unhealthy obesity, with recent studies demonstrating that specific lipid, oxidative, and inflammatory signatures can identify individuals at the highest risk for complications despite similar BMI [140].

## 7. Therapeutic Modulation of Lipid Signaling and Inflammation in Obesity Using Antioxidants

Promising therapeutic targets for obesity management include the interplay between oxidative stress, lipid signaling, and inflammation. Recent evidence demonstrates that antioxidants can modulate these pathways, offering potential therapeutic benefits beyond their traditional free radical scavenging properties (Table 2).

Polyphenols, particularly resveratrol, have emerged as potent modulators of lipid-induced inflammation in adipose tissue. Resveratrol reduces pro-inflammatory cytokine production and macrophage infiltration by inhibiting NF-κB via sirtuin-1 (SIRT1) and by suppressing the NLRP3 inflammasome, while enhancing adiponectin secretion [141,142,143,144]. In individuals with obesity and related pathologies, resveratrol supplementation at doses of 500 mg/day or higher for over 12 weeks has been shown to reduce inflammatory markers like C-reactive protein (CRP) and TNF-α, alongside a decrease in BMI [145]. More recent trials using doses up to 1 g/day have reported pleiotropic effects, including calorie restriction-like responses. While doses ≥ 500 mg/day (especially >1 g/day) were associated with gastrointestinal adverse events (nausea, diarrhea, abdominal discomfort) and potential drug interaction risks via modulation of cytochrome P450 isoenzymes, lower doses were generally well tolerated [146,147].

Similarly, epigallocatechin gallate (EGCG) from green tea disrupts lipid raft formation in macrophages, preventing TLR-4 dimerization and subsequent inflammatory cascade activation in response to dietary lipids [148,149,150,151] (Table 2). Although the effects of EGCG on overall metabolic health in human obesity remain controversial, green tea catechin supplementation has shown moderate benefits on lipid profiles in individuals with overweight and obesity, particularly by lowering triglycerides and increasing high-density lipoprotein cholesterol (HDL-C) [152]. Oral studies have employed up to 1200 mg/day EGCG (600–1200 mg for approximately 4 months) in adult populations, showing modest metabolic or symptom benefits [153]. In addition, pilot studies combining EGCG with other antioxidants, such as β-cryptoxanthin, caffeine, or resveratrol, demonstrated synergistic effects on fat oxidation, gut microbiota modulation, or metabolic pathways in adults with overweight/obesity [154,155,156].

Curcumin restores lipid balance in diet-induced obesity models, leading to inflammatory effects and improved insulin resistance. This is achieved by enhancing the production of anti-inflammatory epoxyeicosatrienoic acids (EETs) through cytochrome P450 epoxygenase, while simultaneously preventing their degradation via the inhibition of soluble epoxide hydrolase [157,158,159]. Moreover, curcumin modulates specialized SPMs derived from omega-3 fatty acids, enhancing the resolution of adipose tissue inflammation [160,161,162] (Table 2). Likewise, the anti-inflammatory effects of curcumin have been consistently observed in individuals with overweight and obesity. Supplementation at doses above 500 mg/day for 8–10 weeks significantly decreases serum levels of inflammatory markers like IL-1β, IL-6, and TNF-α [163,164,165]. Clinical trials have confirmed curcumin’s safety profile at doses up to 1000 mg/day in these populations, with mild gastrointestinal disturbances being the most commonly reported adverse effects [166]. Furthermore, the combination of resveratrol and curcumin has been studied in both cellular and clinical models. While human trials remain limited and show modest postprandial effects, preclinical evidence supports potential additive or synergistic interactions. Further research is needed to clarify the impact of resveratrol and curcumin on anti-inflammatory and antioxidant responses in metabolic diseases [167,168].

Astaxanthin, a xanthophyll carotenoid, exhibits unique membrane-stabilizing properties that prevent lipid peroxidation and inhibit the interaction between OxPLs and CD36 receptors on macrophages, thereby preventing foam cell formation and reducing inflammatory cytokine production [169]. Additionally, astaxanthin enhances PPARγ signaling, promoting adipocyte differentiation toward a healthier metabolic phenotype characterized by improved lipid storage capacity and reduced ectopic lipid deposition [170] (Table 2). A meta-analysis of human trials across various populations, including those with obesity and T2DM, suggests that astaxhanthin supplementation has modest effects on lipid profiles, primarily by increasing HDL-C. In addition, effects on inflammatory markers such as CRP were observed with higher doses (>12 mg/day) for longer durations (>12 weeks) [171]. Astaxanthin is generally well tolerated at these doses, with adverse events limited to rare, mild gastrointestinal symptoms. No severe hepatotoxicity or allergic reactions have been reported in these trials [172].

Quercetin intervention has revealed novel mechanisms involving sphingolipid metabolism that reduce hepatic ceramide accumulation, which is implicated in insulin resistance and inflammatory signaling. Furthermore, quercetin reduces sphingosine-1-phosphate (S1P) production, which counteracts pro-inflammatory cytokine signaling [173,174] (Table 2). Human clinical trials suggest quercetin can benefit obesity and metabolic syndrome components [175]. In individuals with overweight and obesity, quercetin supplementation at doses over 100 mg/day for 12 weeks has been shown to reduce total body fat, BMI, and triacylglycerol. In obese subjects at high cardiovascular risk, the consumption of 150 mg/day of quercetin for 6 weeks reduced systolic blood pressure and low-density lipoprotein cholesterol (LDL-C). Additionally, 250 mg/day of quercetin for 8 weeks improved insulin level, blood glucose, and lipid profiles in individuals with T2DM [175]. Quercetin supplementation is generally safe in humans, with rare adverse effects. However, high-dose, long-term use may pose nephrotoxic risks in individuals with pre-existing kidney conditions, and caution is advised in patients with estrogen-dependent cancers due to potential tumor-promoting effects. Additionally, findings from clinical studies are limited by heterogeneity in study design, dosage, and population [176].

Similarly, anthocyanins modulate lipid raft composition in human endothelial cells, disrupting the assembly of pro-inflammatory signaling platforms [177]. Clinical studies have shown that anthocyanin supplementation may improve metabolic parameters associated with obesity and related disorders. For instance, daily intake of 320 mg anthocyanins from bilberry and blackcurrant extracts for 12 weeks led to a significant reduction in LDL-C and improved insulin sensitivity in hyperlipidemic patients. Additionally, anthocyanin consumption has been linked to enhanced lipid profiles and decreased inflammatory markers, suggesting potential therapeutic benefits in mitigating cardiometabolic risk in individuals with obesity [178]. Anthocyanins are generally well tolerated in clinical studies at doses of 50–320 mg/day, with only occasional mild gastrointestinal symptoms reported. Animal studies support their safety at much higher doses (up to 8000 mg/kg/day), though some sub-acute toxicity has been observed when combined with other compounds, suggesting potential interactions. However, no tolerable upper intake level has been established, and long-term safety data in humans remain limited [179,180].

Sulforaphane, an isothiocyanate from cruciferous vegetables, activated Nrf2, which also regulates lipid metabolism through interaction with PPARγ, but increased adiponectin expression [181]. Additionally, sulforaphane disrupts the crosstalk between oxidized lipids and pattern recognition receptors, attenuating sterile inflammation characteristic of metabolic syndrome [182,183] (Table 2). Recent clinical evidence suggests that sulforaphane may exert beneficial metabolic effects in individuals with obesity. In a placebo-controlled clinical trial, daily administration of a broccoli sprout extract (providing 150 µmol sulforaphane equivalents/day) for 12 weeks significantly reduced fasting blood glucose and glycated hemoglobin (HbA1c) levels in individuals with obesity and T2DM, demonstrating efficacy comparable to metformin [184]. These findings support the potential role of sulforaphane as an adjunct therapeutic strategy for obesity-associated metabolic disorders. Sulforaphane appears generally safe for adults with metabolic risk at doses up to 150 µmol/day, with mild gastrointestinal symptoms being the most reported side effect. Although long-term safety remains unclear, preclinical and in vitro studies combining sulforaphane with other antioxidants or phytochemicals (e.g., curcumin) suggest potential additive or synergistic benefits on oxidative stress and inflammatory biomarkers [185,186,187].

The emerging evidence highlights the multifaceted actions of antioxidants beyond radical scavenging, particularly in modulating lipid-mediated inflammatory signaling. These findings underscore the potential of antioxidant compounds as therapeutic agents for obesity-related metabolic disorders, warranting further clinical investigation to validate their efficacy and establish optimal treatment protocols.

**Table 2:** Therapeutic modulation of lipid signaling and inflammation in obesity using antioxidants.

Antioxidant Compound (Family)	Structure Type	Molecular Targets	Mechanism of Action	References
Resveratrol (Stilbenes/Polyphenols)	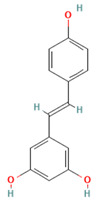	SIRT1, NF-κB, NLRP3 inflammasome	Activates SIRT1 (inhibits NF-κB, enhances adiponectin secretion); suppresses NLRP3 inflammasome activation (attenuates IL-1β, reduces macrophage infiltration)	[141,142,143,144]
Epigallocatechin gallate (EGCG) (Catechins/Polyphenols)	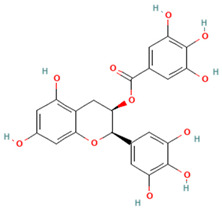	Lipid rafts, TLR-4, NLRP3 inflammasome	Disrupts lipid raft formation in macrophages (prevents TLR-4 dimerization, blocks inflammatory cascade)	[148,149,150,151]
Curcumin (Curcuminoids)	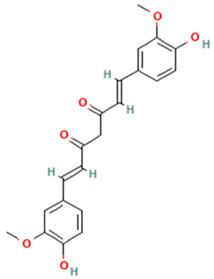	Cytochrome P450, soluble epoxide hydrolase, SPMs	Enhances cytochrome P450 epoxygenase activity (increases EETs); inhibits soluble epoxide hydrolase (inhibits EET degradation); modulates specialized SPMs	[157,158,159,160,161,162]
Astaxanthin (Xanthophyll carotenoids)	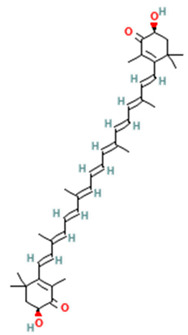	Cell membranes, CD36 receptors, PPARγ	Membrane-stabilizing (prevents lipid peroxidation, OxPLs formation); inhibits interaction between oxidized phospholipids and CD36 receptors; enhances PPARγ signaling	[169,170]
Quercetin (Flavonoids/Polyphenols)	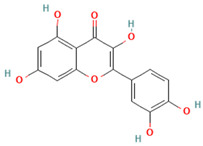	Ceramide synthesis pathway, ceramidase, S1P	Inhibits ceramide synthesis, enhances ceramidase activity (reduces hepatic ceramide); reduces S1P production (counteracts pro-inflammatory signaling)	[173,174]
Anthocyanins (Flavonoids/Polyphenols)	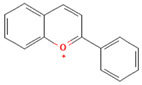	Lipid rafts, inflammatory signaling platforms	Modulate lipid raft composition in human endothelial cells (disrupt pro-inflammatory platforms, enhance anti-inflammatory effect)	[177]
Sulforaphane (Isothiocyanates)	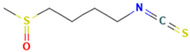	Nrf2, PPARγ, pattern recognition receptors	Activates Nrf2 (induces antioxidant enzymes, regulates lipid metabolism via PPARγ); increases adiponectin expression; suppresses lipogenesis; reduces lipotoxicity; disrupts oxidized lipid-receptor crosstalk	[180,181,182]

## 8. Conclusions

**Figure 3:**
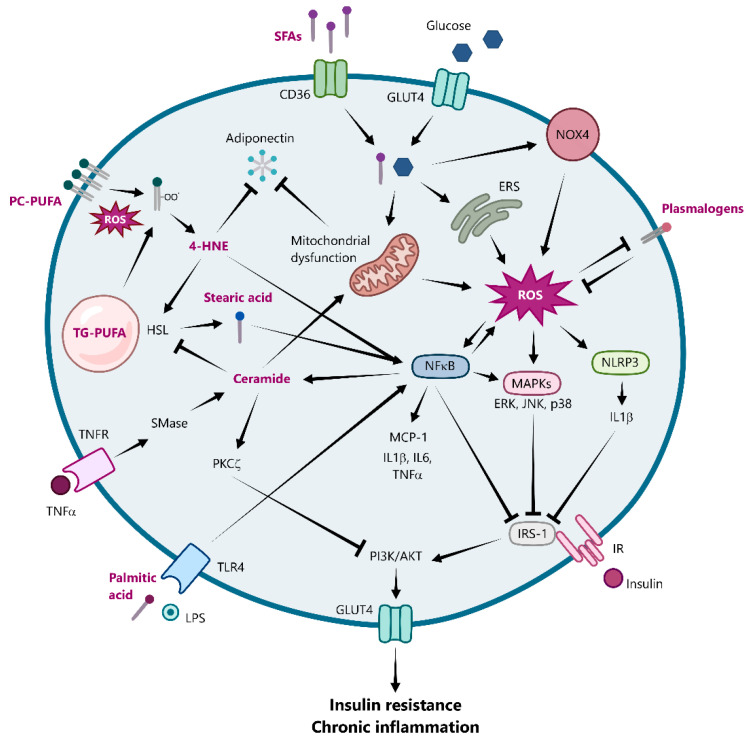
The interplay between oxidative stress, lipid intermediates, inflammation, and insulin resistance in obese adipocytes. In hypertrophic adipocytes, chronic excess of SFAs and glucose induces mitochondrial dysfunction, NOX4 overactivation, and ERE, collectively promoting ROS production. ROS trigger inflammatory signaling pathways, including NF-κB, MAPKs (ERK, JNK, p38), and NLRP3 inflammasome, leading to the increased expression of pro-inflammatory cytokines (IL-1β, IL-6, TNF-α) and chemokines (MCP-1) and impaired insulin signaling through IRS-1 inhibition. These processes contribute to low-grade chronic inflammation and insulin resistance. Additionally, alterations in adipocyte lipid composition, including triglyceride accumulation and increased saturation of membrane phospholipids, enhance lipid peroxidation, generating intermediates such as 4-HNE that activate NF-κB, reduce adiponectin secretion, and stimulate HSL, increasing fatty acid release, which further amplifies inflammation. TNF-α and palmitic acid promote ceramide accumulation via sphingomyelinase activation and TLR4-NF-κB signaling, respectively. Ceramides worsen mitochondrial dysfunction, impair lipolysis, and inhibit insulin signaling via PKCζ. Meanwhile, antioxidant plasmalogens are depleted as oxidative stress overwhelms protective mechanisms, reinforcing metabolic and inflammatory dysfunction. Arrows with a pointed tip indicate activation or induction of the target pathway or molecule, whereas arrows with a flat end indicate inhibition or suppression of the target. 4-HNE, 4-hydroxynonenal; AKT, protein kinase B; CD36, cluster of differentiation 36; ERK, extracellular signal-regulated kinase; ERS, endoplasmic reticulum stress; GLUT4, glucose transporter type 4; HSL, hormone-sensitive lipase; IL, interleukin; IR, insulin receptor; IRS-1, insulin receptor substrate-1; JNK, c-Jun N-terminal kinase; LPS, lipopolysaccharide; MAPK, mitogen-activated protein kinase; MCP-1, monocyte chemoattractant protein-1; NF-κB, nuclear factor kappa B; NOX4, NADPH oxidase 4; NLRP3, nucleotide-binding domain, leucine-rich-containing family, pyrin domain-containing-3; PC-PUFA, polyunsaturated phosphatidylcholine; PI3K, phosphoinositide 3-kinase; PKCζ, protein kinase C zeta; ROS, reactive oxygen species; SFAs, saturated fatty acids; Smase, sphingomyelinase; TG-PUFA, polyunsaturated triglyceride; TLR4, Toll-like receptor 4; TNF-α, tumor necrosis factor-alpha.

In conclusion, this review has underscored the intricate and multifaceted relationship between lipid metabolism, oxidative stress, and inflammation as key drivers of adipose tissue dysfunction in obesity (Figure 3). The pathological dysregulation of TAG storage leads to adipocyte hypertrophy, which, coupled with an altered redox environment characterized by ROS, fuels a vicious cycle of chronic inflammation and insulin resistance. Specifically, the aberrant generation of bioactive lipid mediators such as ceramides, often driven by increased SFA availability and pro-inflammatory cytokines like TNF-α, directly impairs mitochondrial function and insulin signaling, further exacerbating metabolic complications.

Lipidomic profiling emerges as a powerful tool for identifying specific biomarkers of this dysfunction, including elevated ceramide species, altered phospholipid profiles (such as reduced plasmalogen levels in later stages), and increased lipid peroxidation products. Given its ability to detect subtle metabolic alterations before clinical symptoms appear, lipidomics holds strong potential for the early diagnosis of obesity. Futures studies should further explore and validate specific lipid signatures that can serve as early diagnostic markers, facilitating timely intervention and personalized therapeutic strategies.

Furthermore, antioxidant therapies present a promising avenue for mitigating the detrimental effects associated with obesity by potentially modulating downstream inflammatory signaling pathways (e.g., NF-κB or NLRP3 inflammasome), influencing lipid metabolism enzymes (like those involved in ceramide synthesis or breakdown), and restoring redox-sensitive cellular signaling cascades. Understanding and targeting this complex interplay at multiple levels offers significant opportunities for the discovery and development of new, more specific and selective drugs aimed at addressing adipose tissue dysfunction and preventing the progression to severe metabolic complications such as cardiovascular diseases and T2DM.

## Data Availability

No new data were created or analyzed in this review. The findings are based on the synthesis of existing published literature.

## Abbreviations

The following abbreviations are used in this manuscript:

4-HNE	4-Hydroxynonenal
AGE	Advanced glycation end product
Akt/PKB	Protein kinase B
AMPK	AMP-activated protein kinase
ATGL	Adipose triglyceride lipase
ATMs	Adipose tissue macrophages
BMI	Body mass index
C/EBP	CCAAT/enhancer-binding protein
CAT	Catalase
CHOP	C/EBP homologous protein
CRP	C-reactive protein
DNL	De novo lipogenesis
ER	Endoplasmic reticulum
ERK	Extracellular signal-regulated kinase
GPX	Glutathione peroxidase
GSH	Reduced glutathione
GSSG	Oxidized glutathione
GLUT4	Glucose transporter type 4
H_2_O_2_	Hydrogen peroxide
HDL	High-density lipoprotein cholesterol
HSL	Hormone-sensitive lipase
IκBα	Inhibitor of kappa B alpha
IKK	IκB kinase
IL-6	Interleukin-6
IRS	Insulin receptor substrate
JNK	c-Jun N-terminal kinases
LPS	Lipopolysaccharide
MAPK	Mitogen-activated protein kinase
MCP-1	Monocyte chemoattractant protein-1
NADPH	Nicotinamide adenine dinucleotide phosphate
NF-κB	Nuclear factor kappa-light-chain-enhancer of activated B cells
NLRP3	Nucleotide-binding domain leucine-rich-containing family pyrin domain-containing-3
NOX	NADPH oxidase
Nrf2	Nuclear factor erythroid 2-related factor 2
OxPL	Oxidized phospholipid
PKA	Protein kinase A
PKC	Protein kinase C
PI3K	Phosphoinositide 3-kinase
PPAR	Peroxisome proliferator-activated receptor
PTP	Protein tyrosine phosphatase
PUFA	Polyunsaturated fatty acid
ROS	Reactive oxygen species
SFAs	Saturated fatty acids
SOD	Superoxide dismutase
SPM	Specialized pro-resolving mediator
SREBP1	Sterol regulatory element-binding protein 1
SVF	Stromal-vascular fraction
TAGs	Triacylglycerol
T2DM	Type 2 diabetes mellitus
TNF-α	Tumor necrosis factor-alpha
OH	Hydroxyl radical
O_2_·^−^	Superoxide anion
